# A Novel Bayesian General Medical Diagnostic Assistant Achieves Superior Accuracy With Sparse History: A Performance Comparison of 7 Online Diagnostic Aids and Physicians

**DOI:** 10.3389/frai.2022.727486

**Published:** 2022-07-22

**Authors:** Alicia M. Jones, Daniel R. Jones

**Affiliations:** Eureka Clinical Computing, Eureka Springs, AR, United States

**Keywords:** Bayesian medical diagnosis, symptom checkers, general medical diagnostic assistant, diagnostic performance, Bayesian network, comparison of physicians with AI decision support, AI medical diagnosis, diagnostic decision support system

## Abstract

Online AI symptom checkers and diagnostic assistants (DAs) have tremendous potential to reduce misdiagnosis and cost, while increasing the quality, convenience, and availability of healthcare, but only if they can perform with high accuracy. We introduce a novel Bayesian DA designed to improve diagnostic accuracy by addressing key weaknesses of Bayesian Network implementations for clinical diagnosis. We compare the performance of our prototype DA (MidasMed) to that of physicians and six other publicly accessible DAs (Ada, Babylon, Buoy, Isabel, Symptomate, and WebMD) using a set of 30 publicly available case vignettes, and using only sparse history (no exam findings or tests). Our results demonstrate superior performance of the MidasMed DA, with the correct diagnosis being the top ranked disorder in 93% of cases, and in the top 3 in 96% of cases.

## Introduction

Online AI symptom checkers and diagnostic assistants (DAs) have tremendous potential to reduce misdiagnosis and cost, while increasing the quality, convenience, and availability of healthcare, but only if they can perform with high accuracy (Millenson et al., 2018; Van Veen et al., 2019; Rowland et al., 2020). Machine Learning (ML) and Bayesian Networks (BNs) are promising technologies in healthcare, but both have limitations for general medical diagnosis. Despite major advances in the application of ML to narrow biomedical applications (Beede et al., 2020; Liu et al., 2020; McKinney et al., 2020), challenges remain for its application to *general* medical diagnosis, including the inability to model causal inference (Velikova et al., 2014; Richens et al., 2020), semantic relationships including subtypes (“is-a” and “part-of”), logic, and heuristics; and lack of interpretability. Furthermore, challenges remain in training or educating DAs with electronic medical record (EMR) data, including proper interpretation of incomplete or missing data (Nikovski, 2000), unreliable labels and label leakage, bias (Ghassemi et al., 2020), and the fact that EMRs are designed to document and support care and reimbursement and to minimize legal risks, rather than to describe disorders.

The use of Bayesian approaches for medical diagnosis is well-documented, from early expert systems (Yu et al., 1988; Shwe et al., 1990; Barnett et al., 1998) to today's chatbot triage and symptom checkers (Zagorecki et al., 2013; Baker et al., 2020). But thus far they have fallen short of the desired accuracy despite incremental improvements (Lemmer and Gossink, 2004; Antonucci, 2011; Richens et al., 2020). In previous studies such DAs have underperformed physicians in diagnostic accuracy (Semigran et al., 2015, 2016; Millenson et al., 2018; Chambers et al., 2019; Yu et al., 2019). For example, Semigran et al. (2015) evaluated the performance of 23 symptom checkers using case vignettes, and found they ranked the correct diagnosis first 34% of the time, and in the top 3 in 51% of cases. In a subsequent paper (Semigran et al., 2016) compared symptom checkers to physicians, and showed much better performance for physicians, who ranked the target diagnosis #1 in 72.1% of cases, vs. only 34% for the symptom checkers. A more recent paper (Baker et al., 2020), using 30 of the case vignettes tested in Semigran et al. (2015) and Semigran et al. (2016), reported performance comparable to physicians: the Babylon system ranked the target diagnosis #1 for 70% of the vignettes and in the top 3 for 96.7%, compared to 75.3 and 90.3%, respectively, for physicians. But even the benchmark of obtaining physician diagnostic accuracy leaves much to be desired, with reported physician diagnostic error rates of 10–24% or greater (Graber, 2012; Meyer et al., 2013; Baker et al., 2020). Diagnostic errors are the leading cause of paid malpractice claims (28.6%), and are responsible for the highest proportion of total payments (35.2%) (Tehrani et al., 2013). Diagnostic errors were almost twice as likely to be associated with patient death as other types of errors (e.g., treatment, surgery, medication, or obstetrics errors). Almost 70% of diagnostic errors occurred in the outpatient setting (Tehrani et al., 2013).

BNs model causal inference using Bayes' theorem. They offer a formal method for representing an evolving process of refining the posterior probabilities of outcomes based on the likelihood of relevant data. This approach is particularly suitable for diagnosis, where clinicians formulate an initial differential diagnosis based on the patient chief complaint, and then proceed to refine the diagnosis based on additional data obtained from the patient interview, exams, tests, and treatment outcomes. In this iterative process, each differential diagnosis ranks the likelihood of each contending disorder, and provides priorities for the next data items to ascertain.

Given a joint random variable ***X*** = *X*_1_, …, *X*_*N*_, a Bayesian Network (BN) offers a compact representation of its local conditional probability distributions (Koller and Friedman, 2009). Formally, a Bayesian Network is defined as a pair BN = (*G, P*), where *G* is a directed acyclic graph (DAG) and *P* is the joint probability distribution of ***X*** as specified by the conditional probability tables (CPTs) of the graph nodes. The graph *G* = (*V, E*), is comprised of nodes or vertices *V* and directed arcs or edges *E* ⊆ *V* × *V*. Each node in *V* represents a distinct random variable in ***X***, and each arc in *E* represents the conditional probability of the child node given its parent. Every node is conditionally independent of its non-parent non-descendants, given its parents. It follows that the joint probability distribution *P*(***X***) reduces to the product of the conditional probability distributions at each node (local Markov property), and can be written as:


(1) $$\begin{array}{l} P\left(x_{1},\ldots ,x_{N}\right)=\overset{N}{\underset{i=1}{\prod }}P\left(x_{i}|\pi_{i}\right) \end{array}$$

where π_*i*_ is the state of the joint variable defined by the elements of ***X*** that are the parents of *X*_*i*_ (Fagiuoli and Zaffalon, 1998; Antonucci, 2011).

The size of the CPT describing the joint probability distribution at a node grows exponentially with the number of inputs (parents). For problems involving a large number of variables and/or dense graphs, computational complexity and/or lack of sufficient data can make this approach impractical. The leaky noisy-OR function (Henrion, 1987; Antonucci, 2011) is a popular technique for reducing the input parameter requirements from exponential to linear (for binary variables). It does so by assuming the parent nodes are conditionally independent given their joint child. With this assumption, the joint probability distribution of the child node simplifies to:


(2) $$\begin{array}{l} P\left(x_{i}|\pi_{i}\right)=1-\left(1-n_{i}\right)\underset{x_{j}\in \pi_{i}}{\prod }{\left(1-P\left(x_{i}|x_{j}\right)\right)}^{\delta_{j}} \end{array}$$

where *P*(*x*_*i*_|*x*_*j*_) is the conditional probability of the child node given parent *X*_*j*_, and δ_*j*_ = 1 if *x*_*j*_ = *true* and 0 if it is *false*. Equation (2) can be interpreted as meaning that *X*_*j*_ only affects change when it is present. Ignoring the (1 − *n*_*i*_) term for a moment, we see this is simply the probability formula for the union of independent events, i.e., *P*(⋃_*i*_*A*_*i*_) = 1 − ∏_*i*_((1 − *P*(*A*_*i*_)). The variable *n*_*i*_ is a noise term, which is optionally a function of *X*_*i*_, and represents unmodeled causes of *X*_*i*_ assumed to be present.

A classifier can be defined in conjunction with a BN by assigning each node to 1 of 3 types: (1) input, data, features, or evidence; (2) outputs or class labels; and optionally (3) intermediate or hidden nodes. Given *K* possible outputs, *y*_1_, …, *y*_*K*_, and *L* inputs, *x*_1_, …, *x*_*L*_, the classifier selects the output node *ŷ* such that


(3) $$\begin{array}{l} ŷ=argmax_{i\in \left\{1,\ldots ,K\right\}}P\left(y_{i}|x_{1},\ldots ,\;x_{L}\right) \end{array}$$

where *argmax* selects the maximum argument, i.e., the output node that maximizes *P*(*y*_*i*_|*x*_1_, …, *x*_*L*_). Using Bayes Theorem and assuming the output nodes are mutually independent, Equation (3) reduces to


(4) $$\begin{array}{l} ŷ=argmax_{i\in \left\{1,\ldots ,K\right\}}P\left(y_{i}\right)\cdot P\left(x_{1},\ldots ,\;x_{L}|y_{i}\right) \end{array}$$

where *P*(*y*_*i*_) is the a priori probability of *y*_*i*_. In the special case where the variables *X*_*i*_ are independent, we obtain the naïve Bayes classifier (Koller and Friedman, 2009)


(5) $$\begin{array}{l} ŷ=argmax_{i\in \left\{1,\ldots ,K\right\}}P\left(y_{i}\right)\cdot \overset{L}{\underset{j\;=\;1}{\prod }}P\left(x_{j}|y_{i}\right) \end{array}$$

It is important to keep in mind the assumptions that lead to the simplifications of Equations (4) and (5). Medical diagnosis is one domain in which these assumptions are not always valid, resulting in excessively degraded classification.

**Figure 1:**
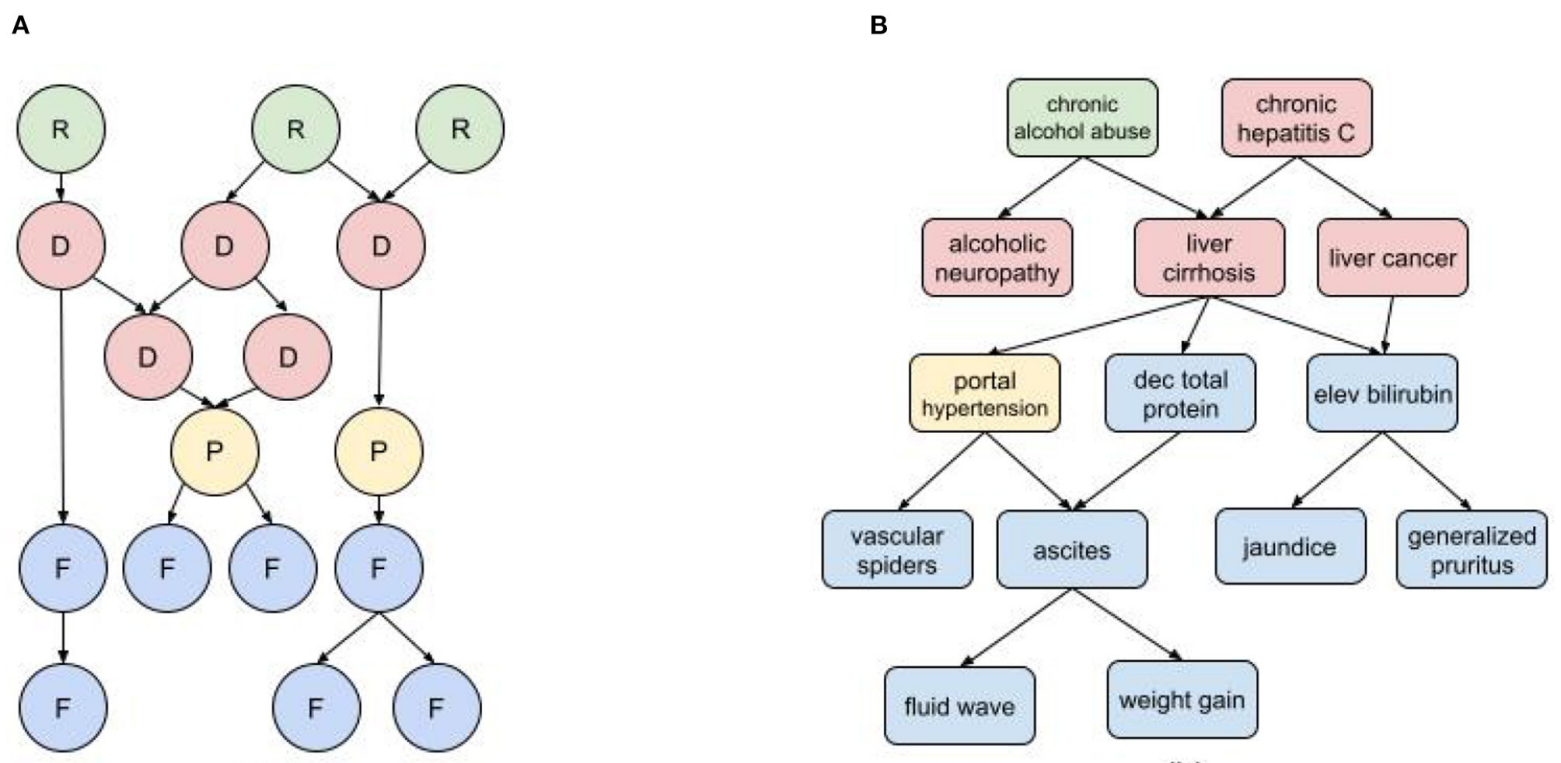
Diagnostic BN hierarchy **(A)** Generic fragment where each node represents a risk factor (R) disease (D), pathophysiological state (P), or findings (F); **(B)** BN fragment for liver cirrhosis.

In a medical diagnostic BN (Figure 1) the input nodes represent all known risk factors and findings (i.e., symptoms, examination results, and test results), while the output nodes are all possible diagnoses. There may also be intermediate nodes representing pathophysiological states or mechanisms. As indicated by the causal arrows, risk factors increase the likelihood of diseases; diseases cause other diseases, pathophysiological states, and findings; physiological states cause findings (and sometimes other physiological states); and findings may cause other findings. For a given set of patient inputs we want to determine the most probable diagnoses using both forward and backward inference.

The characterization of nodes as risk factors, findings, pathophysiological states, and disorders can be governed by somewhat arbitrary nosological distinctions. For example, dehydration is a pathophysiological state with multiple findings (e.g., decreased urine output, dry mucus membranes, dizziness, hypotension), and can be caused by multiple disorders such as acute gastroenteritis and uncontrolled diabetes. But dehydration is also used as a diagnosis when other causal disorders are ruled out, and it can be attributed to, e.g., prolonged exertion in heat without sufficient hydration (a risk factor). The findings of dehydration can be attributed to its causal disorders, but they tend to cluster as a distinct subpopulation in patients with the causal disorders that develop dehydration. The distinction between risk factors and findings can also be ambiguous. For example, obesity is both a risk factor for developing type II diabetes and also a finding of diabetes and other metabolic disorders. And while some findings can cause other findings, it's important not to confuse temporal progression with causality. For example, in an infectious disorder, fever may precede a rash, but doesn't cause it.

**Figure 2:**
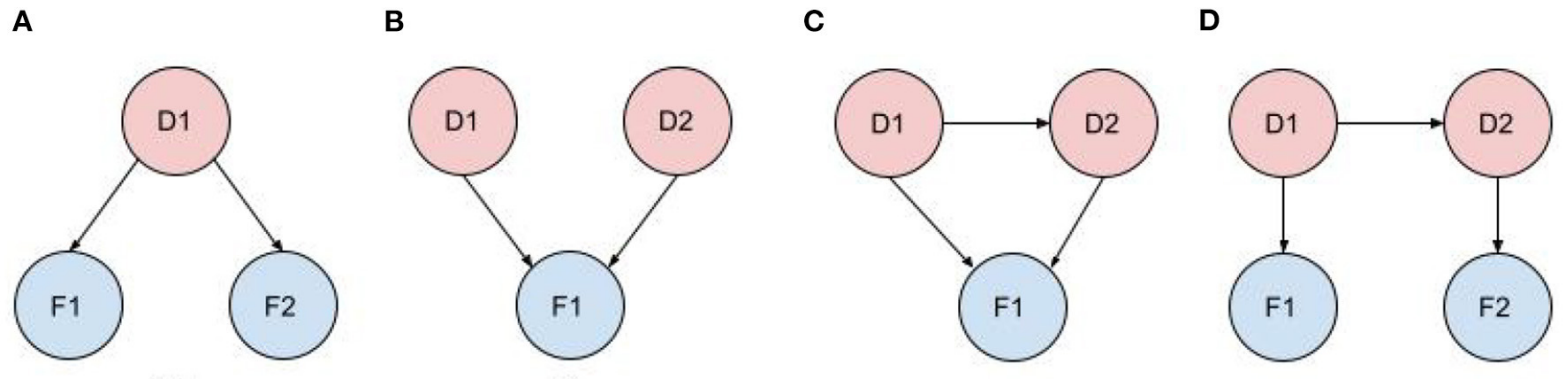
Typical diagnostic BN configurations. **(A)** A disorder causes 2 findings; **(B)** Independent disorders both cause a finding; **(C)** Causally related disorders cause the same finding; **(D)** Causally related disorders each explain a subset of the patient findings.

Figure 2 shows typical diagnostic BN configurations. In Figure 2A a disorder causes 2 findings (*F*_1_, *F*_2_). These findings may be considered conditionally independent, as in pulmonary embolus (PE) causes cough and syncope (the 2 symptoms result from distinct pathophysiologic pathways); or they may be conditionally dependent, as in pulmonary embolus causes cyanosis and syncope (both result from a common pathophysiologic pathway of a PE subset, massive embolism causing circulatory obstruction). In Figure 2B two marginally independent disorders cause a single finding, e.g., pneumonia and acute bronchitis both cause cough. In Figure 2C, two causally related disorders each cause the same finding, e.g., chronic hepatitis causes cirrhosis and both disorders cause hyperbilirubinemia and jaundice; or acute bronchitis precipitates a COPD flare and both cause cough. In Figure 2D, two causally related disorders each explain a distinct subset of the patient findings, e.g., deep vein thrombosis causes pulmonary embolus, with patient findings leg edema (caused by DVT) and dyspnea (caused by PE).

BNs have been a popular choice for medical diagnosis because of their ability to model complex domains and to provide a sound basis for their inference. Compared to pure ML solutions, BNs can incorporate derived medical knowledge (e.g., published studies, textbooks, expert opinion), and do not require huge raw datasets. Fundamental problems with traditional Bayesian implementations include:

- Severe scalability problems due to the large number of nodes required for a diagnostic network with a large number of diagnoses and/or findings (Cheng and Druzdzel, 2000; Heckerman, 2013). A general medical diagnosis BN (e.g., for primary care) may have thousands of diagnoses and tens of thousands of findings. The richer the model, the larger and more complex the DAG becomes, and the more data is required to populate the CPTs. Furthermore, high accuracy requires that many of the findings be modeled as continuous or categorical random variables which can make the CPTs very large.
- Inability to model large-scale knowledge representations (Koller and Pfeffer, 1997). The BN DAG represents a single semantic dimension (causality), but other relationships are required to represent the diagnostic process. Of specific interest in diagnosis is the ability to model inheritance hierarchies. For example, to diagnose “brain tumor or neoplasm” or one of its many subtypes, a conventional BN would require the parent disorder and all of its descendants to each independently be represented in the DAG. This presents not only complexity issues but also defies basic diagnostic heuristics, e.g., that “brain tumor” shouldn't “compete” in the differential diagnosis with its child, “dominant temporal lobe tumor”.
- Failure to capture the semantic overlap or partial synonymy among findings. Semantic overlap is an inevitable byproduct of a complex ontology. When semantic overlap occurs, findings cannot be considered independent, and they jointly fail to deliver the same diagnostic power that is implied by the assumption of independence. For example, if a chest x-ray shows left atrial enlargement (LAE), then an echocardiogram showing LAE may provide slightly more information since it has a higher specificity, but not as much as if the x-ray had not been discerned. Similarly, if we first discerned that the echocardiogram shows LAE, then the x-ray has little to no additional diagnostic value. The effect of semantic overlap in a system that assumes findings are independent can cause overconfidence or premature closure, leading the system to conclude that a specific disease is the correct diagnosis when in fact there is insufficient evidence for that claim. One approach that has been proposed to partially address this problem is to introduce an intermediate node that represents the collective effect of a set of correlated findings (Yu et al., 1988; Nikovski, 2000; Velikova et al., 2014).
- Failure to capture higher order statistics among finding nodes of a given disorder, e.g., how findings vary with duration of symptoms, age, gender, and other risk factors. For example, gender *per se* has little effect on the likelihood of psoriatic arthritis (PA), but males with PA are significantly more likely to present with involvement of a single joint.
- Failure to capture causal relationships among disorder nodes (Richens et al., 2020). The assumption that a patient's findings must be explained by a single disorder rather than the simultaneous occurrence of multiple causally linked disorders can cause underconfidence (diffidence), leading the system to fail to rank the correct diagnosis or diagnoses as the top disorder(s) even after sufficient information was presented for that claim. For an in-depth discussion of diffidence and over-confidence detection in diagnostic systems, see (Hilden et al., 1978).

## Materials and Methods

This paper describes the MidasMed DA, a prototype system based on a novel BN with improved diagnostic modeling. A comprehensive description of the diagnostic engine that powers the MidasMed DA is outside of the scope of this paper. However, we provide highlights of the solution architecture and key innovations that address the fundamental limitations of traditional implementations listed above, and advance the state-of-the-art in AI diagnosis.

The solution architecture consists of the following key components:

- A rich semantic model that captures entity data and relationships among entities of the medical ontology that is largely independent of implementation constraints. The semantic model is instantiated as an object-oriented model for efficient diagnostic computations.
- A diagnostic engine that for each diagnostic request dynamically generates a sparse BN, and then applies a Bayesian classifier to generate a differential diagnosis. The classifier implements disorder subtype hierarchies to recursively and efficiently generate a differential diagnosis with the maximum disorder specificity supported by the data. For example, if warranted by the data, the system will report “anteroseptal acute myocardial infarction” instead of the less specific “acute myocardial infarction.” Note that for many disorders, optimum treatment depends on knowing the specific subtype.
- A “Best Next Finding” module that generates a set of additional findings to discern (from the patient or clinician) in order to most quickly and economically refine the diagnosis.

The semantic model describes the medical ontology and the relationships among its concepts using statistical, logical, and heuristic data. The model can be edited and viewed using a web-based content management system (CMS), and is stored in a semantic SQL database. A constructor algorithm generates an object-oriented model from the semantic assertions in the database, resulting in a Data Transfer Object (DTO). The DTO may be serialized for storage and transport to the server running the diagnostic engine. The DTO represents an in-memory object-oriented image of the semantic model that enables rapid and efficient diagnostic computation in real-time. The DTO abstractly represents the global BN, although other (more efficient) data structures are used to hold the node objects. Each node encapsulates all the information it needs to discover its graph neighbors *via* pointers to other nodes.

Our diagnostic model focuses primarily on the following aspects: (1) dependencies among disorders, (2) subtype relations within a disorder family, (3) the characterization of each disorder in terms of its relevant findings and risk factors, (4) statistical correlation and semantic overlap among findings, and (5) finding contingency hierarchies stemming from the relative semantic scope of each finding and the linear progression of the diagnostic interview. Each of these topics in described in the following sections.

### Inter-disorder Dependency

Disorder dependency is important to model because a patient may present with symptoms of both a causal disease and its complication(s). For example, a patient might present with deep venous thrombosis (DVT) in a leg, combined with symptoms of pulmonary embolus, a life-threatening complication of DVT. In cases where the initial cause is insidious or insufficiently bothersome, or when the cause and its complication(s) occur in rapid succession, the causal disorder may not have been previously diagnosed. We do not want the classifier to “punish” a disorder for not explaining findings of its co-presenting dependent disorder(s); rather, such combinations of findings often provide high confidence for the diagnosis of a *combination* of causally linked disorders. Therefore, our classifier is designed to identify single disorders or clusters of dependent disorders that best explain the patient findings. Of course two *independent* disorders may also jointly explain the patient findings; however, the probability of such an event is generally much lower.

We used the term *Multi-Disease Model* (MDM) to describe a classifier that detects and accounts for clusters of dependent disorders in the differential diagnosis. One of the consequences of MDM is that co-occurring dependent disorders may each explain some of the same finding(s). We therefor need a mechanism for describing how the joint interaction among disorders affects the presentation of their common findings. We use the term *equivalent sensitivity* to describe the sensitivity of a finding that is relevant to multiple dependent disorders that are all assumed to be present (with appropriate extensions for categorical and continuous findings). To illustrate this case, suppose *D*_1_ causes *D*_2_, and both share common a finding *F*_1_ with sensitivities *s*_1,1_ = *P*(*F*_1_|*D*_1_) and *s*_1,2_ = *P*(*F*_1_|*D*_2_). The cluster consisting of *D*_1_ and *D*_2_ has 3 configuration: {$D_{1}^{+}$, $D_{2}^{-}$}, {$D_{1}^{-}$, $D_{2}^{+}$}, and {$D_{1}^{+}$, $D_{2}^{+}$}, where the +/– indicate whether the disorder is present or absent. When both disorders are present, *F*_1_ will have an equivalent sensitivity for the configuration that depends on (a) the nature of *F*_1_, (b) the sensitivities *s*_1,1_ and *s*_1,2_, and (c) whether or not *F*_1_ arises in *D*_1_ and *D*_2_ due to shared or distinct pathophysiological mechanisms. For example, if *F*_1_ is body temperature, *D*_1_ causes hypothermia and *D*_2_ causes fever (an admittedly unusual case), then we would expect the patient temperature (given that she has both *D*_1_ and *D*_2_) to be $s_{1,1}<{\bar{s}}_{1}<s_{1,2}$. On the other hand, if *D*_1_ and *D*_2_ both cause fever, and due to the same underlying mechanism, then we expect ${\bar{s}}_{1,}\approx \;\max(s_{1,1},\;s_{1,2})$. But if *D*_1_ and *D*_2_ both cause fever due to different mechanisms, we might expect ${\bar{s}}_{1}>\;\max(s_{1,1},\;s_{1,2})$. Now suppose *F*_1_ is *time to diagnosis*, with the corresponding question “How long ago did your symptom(s) begin?”. If *D*_1_ has a gradual onset with a distribution centered on “months to years”, while *D*_2_ has a shorter onset, say “days to weeks” then the equivalent sensitivity will satisfy ${\bar{s}}_{1}\approx \;\max(s_{1,1},\;s_{1,2})$, because the patient will most likely associate the beginning of the problem with the onset of *D*_1_, which started first.

To formally describe MDM, consider a cluster of dependent disorders. To qualify, each cluster member must have at least one link to another cluster member, and must explain at least one abnormal patient finding. A disorder may belong to at most one cluster, for if it belonged to multiple clusters those would be merged into a single cluster. A disorder with no dependencies is called a *singleton* (cluster of size 1). Let *D*_1_, …, *D*_*N*_ be members of cluster *C*, and *F*_1_, …, *F*_*M*_ be the known patient findings. The *configurations* of *C* are all permutations of the cluster disorders in which some are present and others are absent. For the net probability of *C* (all configurations) we have:


(6) $$\begin{array}{l} P\left(C|f_{1},\ldots ,f_{M}\right)=\underset{j}{\sum }P\left(C_{j}^{+}|f_{1},\ldots ,f_{M}\right)=\underset{j}{\sum }I\left(C_{j}^{+}\right)\cdot \overset{M}{\underset{i\;=\;1}{\prod }}{\bar{s}}_{ij} \end{array}$$

where $I(C_{j}^{+})$ is the joint incidence (prior probability) of the disorders in $C_{j}^{+}$ co-occurring, and ${\bar{s}}_{ij}$ is the equivalent sensitivity for finding *F*_*i*_ in $C_{j}^{+}$. The probability of cluster disorder *D*_*k*_ is the sum of the probabilities of all configurations in which it is present, i.e.,


(7) $$\begin{array}{l} P\left(D_{k}|f_{1},\ldots ,f_{M}\right)=\underset{j}{\sum }P\left(C_{j}^{+}|f_{1},\ldots ,f_{M}\right)\cdot \delta_{jk} \end{array}$$

where ${\;\delta }_{jk}=1\;if\;D_{k}\in C_{j}^{+}$ and 0 otherwise. While the total number of configurations may be very large (since *C* may be large) this does not present a computational problem, since the vast majority of configurations can be discarded using pruning heuristics with negligible effect on the accuracy of the cluster probability computation. Note that given the set of all contending diagnoses across all clusters, the cluster probabilities sum up to 1.0 but the disorder probabilities do not, due to co-occurrence among the disorders.

### Disorder Subtype Hierarchies

The ability to model disorder subtypes is important in diagnosis, because disorder subtypes may have different prognoses and/or require different treatments (e.g., viral vs. bacterial meningitis). We use the term *subtypy* to define a framework for describing the disorder inheritance hierarchy. Note that inheritance hierarchies in diagnosis are statistical and not directly analogous to the programming concept of object-oriented inheritance. In diagnosis, the ancestor represents a statistical aggregate of its descendants or variants, and while it may be convenient to think of a subset of findings as manifest in the parent and passed on to the children, there are usually variations in how these findings are expressed (or not) in each child. For example, conjunctival injection is always present in infectious conjunctivitis, and inherited to both subtypes gonococcal (bacterial) conjunctivitis and viral conjunctivitis. However, conjunctival hemorrhages are more common in the viral variant, while eyelid edema and purulent discharge are more common the bacterial variant. Furthermore, a Gram stain of the gonococcal conjunctivitis discharge may identify Gram-negative diplococci, but it is irrelevant to the viral variant. So the Gram stain test finding is relevant to the parent (infectious conjunctivitis), but not to its viral child. In summary, a child attribute is always represented by the parent, but not necessarily vice versa, and the manifestation in the parent is a statistical aggregate of its children.

Because each parent represents the statistical aggregate of its children, and the probability of each child varies based on the patient findings, we must compute all sensitivities dynamically for each new set of patient findings, and we must do so by starting at the very bottom of the hierarchy tree (the “leaves” or childless disorders). To see why this is the case, consider a simple example with parent disorder meningitis and its children viral and bacterial meningitis. The prior probability (incidence) of meningitis in the U.S. is ~9.25e-5. Approximately 82% of cases are viral and 18% bacterial. Consider the finding “CSF culture positive for bacteria.” This finding is relevant to bacterial meningitis with *s*_*bm*_ ≈0.95 and is not relevant to viral meningitis, so we assign a noise sensitivity, e.g., *s*_*vm*_=0.02, and compute the sensitivity in the parent as the weighted sum: *s*_*m*_ = (*I*_*vm*_ · *s*_*vm*_ + *I*_*bm*_ · *s*_*bm*_)/*I*_*m*_ = 0.82 · 0.02 + 0.18 · 0.95 = 0.187. Now suppose this finding was determined to be positive in the patient. The posterior relative probability of the children is now *P*_*vm*_ = *I*_*vm*_ · *s*_*vm*_ = 0.82 · 9.25e-5 · 0.02 = 1.152e-6 and *P*_*bm*_ = *I*_*bm*_ · *s*_*bm*_ = 0.18 · 9.25e-5 · 0.95 = 1.58e-5. The relative probability of the children has changed from 0.82/0.18 to 0.07/0.93, and *s*_*m*_ = 0.07 · 0.02 + 0.93 · 0.95 = 0.88. Similarly, if the finding was negative in the patient then *P*_*vm*_ = *I*_*vm*_ · (1 − *s*_*vm*_) = 0.82 · 9.25e-5 · 0.98 = 7.43e-5, *P*_*bm*_ = *I*_*bm*_ · (1 − *s*_*bm*_) = 0.18 · 9.25e-5 · 0.05 = 8.32e-7, the relative probability ratio is 0.99/0.01 and *s*_*m*_ = 0.99 · 0.02 + 0.01 · 0.95 = 0.03.

From the end user perspective it is desirable for the diagnostic process to proceed from the general to the specific (e.g., from “stoke or TIA” to “cortical posterior cerebral artery stroke, dominant”) progressively as more of the relevant patient findings are discerned. To do so, we use a heuristic called *Child Better than Next* that replaces a parent disorder by all its direct children provided that the relative probability of at least one of the children exceeds that of the next disorder in the differential diagnosis stack. This requires the disorders to be ranked by descending relative probability, and for the stack to be resorted after each replacement.

### Disorder Findings Dependencies

Each finding is modeled as binary, discrete multi-valued (categorical), or a continuous random variable. We use the term “finding” broadly to include risk factors, and distinguish between them by selecting the appropriate interaction model (e.g., reflecting direction of causality) when computing their impact on disorder probabilities.

**Figure 3:**
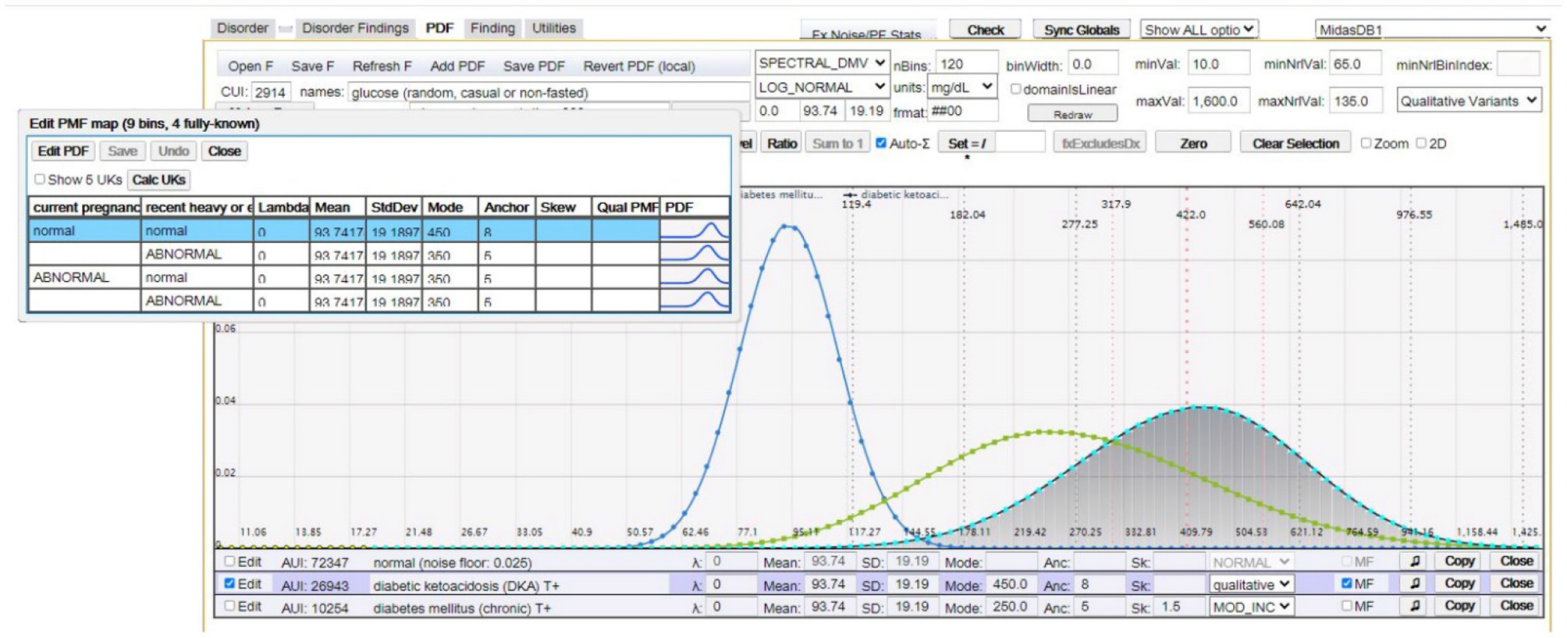
This figure (image captured from our CMS) shows random serum glucose modeled as a log normal distribution for (peak distributions left-to-right): normal (healthy), chronic diabetes mellitus, and DKA. The overlay table in the top left shows *multifactorial* distributions of serum glucose for DKA as a function of factor findings “current pregnancy” and “recent heavy alcohol consumption”.

While some findings may justifiably be modeled as conditionally independent for a given disorder (Naïve Bayes), this is not the case in general. Frequently, findings vary with other findings that are not directly relevant to the index disorder. In such cases we can write:


(8) $$\begin{array}{l} P\left(f_{1}|D\right)=P\left(f_{1}|f_{2},\ldots ,\;f_{L},D\right) \end{array}$$

where *F*_1_ is relevant to *D* and *P*(*f*_1_|*D*) can be described by a multidimensional probability distribution, with *factor findings F*_2_, …, *F*_*L*_ that are not necessarily directly relevant to *D*, but act as factors in the computation of its finding probabilities. Common factor findings are age, gender, and time-to-diagnosis; however, many findings have their unique factor findings. For example, Figure 3 depicts the distribution of serum glucose for diabetic ketoacidosis (DKA) as a function of factor findings “current pregnancy” and “recent heavy alcohol consumption”.

### Inter-findings Dependencies

Failure to capture semantic overlap or disjunction can cause significant distortion unless inter-finding dependencies are properly managed. At the root of the problem is the basic concept of finding *diagnostic power*. The diagnostic power of a finding represents how much information it contributes to the likelihood of a disorder relative to contending disorders. That is, given what we already know about the likelihood of a disorder from its prior probability (incidence) and previously ascertained findings, how much *additional* information does a new finding provide? We define diagnostic power using a measure called the *probability factor* (PF), which is the ratio of the probability of the finding in the disorder relative to its prevalence in the general population. Table 4 in Supplementary Materials shows how this measure relates to other popular measures that quantify the discriminating power of a finding.

To illustrate the problem of semantic overlap, consider a patient complaining of pain, edema (swelling), and erythema (redness) at the knee. These findings collectively represent aspects of knee joint inflammation in rheumatoid, traumatic, or reactive arthritis. Note however that these findings are not correlated or even jointly relevant for all disorders that cause knee pain. For example, L4 lumbar disc herniation can cause knee pain, but not edema or erythema.

We address semantic overlap by defining an intermediate node called an *xopathy* (a generalization of terms such as neuropathy, dermopathy, or arthropathy). The xopathy framework enables us to represent a set of findings that are conditionally dependent with respect to an index disorder using an interim aggregate node. The xopathy sensitivity represents the incidence of the xopathy in the population of patients with the disorder. The xopathy sensitivity can also be interpreted as the conditional probability that one or more of the xopathy findings is present given the index disorder.

Let *D* represent a disorder with conditionally *dependent* findings *F*_1_, …, *F*_*L*_. We construct an xopathy *Xop* with the findings as its members, and each having a sensitivity *s*_*i*_ = *P*(*f*_*i*_|*xop*). We are also given the xopathy sensitivity, *s*_*Xop*_ = *P*(*xop*|*D*). Our goal is to compute dynamic sensitivities $s_{1}^{*},\ldots ,\;s_{K}^{*}$, *K* ≤ *L* for each *known* finding that satisfy


(9) $$\begin{array}{l} P\left(f_{1},\ldots ,\;f_{K}|D\right)=\overset{K}{\underset{i=1}{\prod }}s_{i}^{*} \end{array}$$

The actual algorithms for computing $\left\{s_{i}^{*}\right\}$ are beyond the scope of this paper. However, we provide a brief outline of the process with key equations.

**Step 1**: Compute the *independent* xopathy diagnostic power (probability factor), *PF*_*indep*_, as the product of the finding PFs. This represents the diagnostic power we would introduce into the disorder probability computation if we assumed the findings were independent. As noted earlier, *PF*_*indep*_ will generally be greater than the *desired* diagnostic power when the findings are correlated.


(10) $$\begin{array}{l} PF_{indep}\left(Xop\right)=\overset{K}{\underset{i=1}{\prod }}PF\left(f_{i}|Xop\right) \end{array}$$

where (*f*_*i*_|*Xop*) = *s*_*i*_/*n*_*i*_, *n*_*i*_ is the prevalence of *F*_*i*_ in the general population, and *s*_*i*_ is the finding sensitivity relative to the xopathy. Note that the findings are independent relative to the xopathy (but not the disorder), which allows us to use the Naïve Bayes assumption in Equation (10).

**Step 2**: Determine the maximum allowed PF for this xopathy, *PF*_max_(*Xop*). If *PF*_*indep*_ exceeds *PF*_max_ then apply compression to decrease finding sensitivities. We denote the compressed sensitivities $\left\{{\ddot{s}}_{i}\right\}$. The compression algorithm must satisfy several constraints, such as preserving the relative magnitude of the original sensitivities $\left(s_{i}>s_{j\;}\to {\ddot{s}}_{i}>{\ddot{s}}_{j}\right)$, and ensuring that positive findings remain so $\left(\frac{s_{i}}{n_{i}}>1\to \frac{{\ddot{s}}_{i}}{n_{i}}>1\;\right)$.

**Step 3**: Reflect the xopathy sensitivities to the disorder. The sensitivities $\left\{{\ddot{s}}_{i}\right\}$ represent the conditional probability of the findings on the xopathy, but what we really want is sensitivities conditioned on the disorder per Equation (9). Let _*x*_0_ = *sXop*_ = *P*(*xop*|*D*), $ŝ={\left(\prod_{i\;=\;1}^{K}{\ddot{s}}_{i}\right)}^{\frac{1}{K}}$, and $\hat{n}={\left(\prod_{i=1}^{K}n_{i}\right)}^{\frac{1}{K}}$, where ŝ and $\hat{n}$ represent the geometric means of $\left\{{\ddot{s}}_{i}\right\}$ and {*n*_*i*_}, respectively. For simplicity, in this derivation we're interpreting *s*_*i*_ as the probability of the finding *F*_*i*_ in its *known* state. If the finding is negative then *s*_*i*_ = 1 − *P*(*F*_*i*_ *is positive*).

We initialize the algorithm as follows:


(11) $$\{\begin{array}{c} {\tilde{s}}_{1}=x_{0}\cdot \hat{s}+(1-x_{0})\cdot \hat{n}\\ x_{1}=x_{0}\cdot \frac{\hat{s}}{{\tilde{s}}_{1}} \end{array}$$

Note that ${\tilde{s}}_{1}$ is the expected sensitivity over the two mutually exclusive disorder subpopulations: the xopathy population with prior probability *x*_0_, and the complementary population with prior probability (1 − *x*_0_). With each discerned finding, the probability that the patient belongs to the xopathy subpopulation changes. If the finding was positive the xopathy probability increases and if it was negative it decreases.

Similarly, for the remaining iterations, *j* = 2, .., *K* we have:


(12) $$\{\begin{array}{c} {\tilde{s}}_{j}=x_{j-1}\cdot \hat{s}+(1-x_{j-1})\cdot \hat{n}\\ x_{j}=x_{j-1}\cdot \frac{\hat{s}}{{\tilde{s}}_{j}} \end{array}$$

Similar to ŝ, we define $\hat{s}={\left(\prod_{i\;=\;1}^{K}{\tilde{S}}_{i}\right)}^{\frac{1}{K}}$ as the geometric mean of the raw disorder sensitivities computed in Equation (12). Finally, we normalize the $\left\{{\tilde{s}}_{j}\right\}$ using the scaling factor $R=\frac{š}{ŝ}$ in order to preserve the xopathy diagnostic power achieved in Step 2. The final sensitivities $\left\{s_{i}^{*}\right\}$ for Equation (9) are:


(13) $$\{\begin{array}{c} s_{i}^{*}=R\cdot {\ddot{s}}_{i}\;for\;R\leq 1.0\\ s_{i}^{*}=\frac{R\cdot {\ddot{s}}_{i}}{1+{\ddot{s}}_{i}(R-1)}\;for\;R\;>1 \end{array}$$

The second form of $s_{i}^{*}$ in Equation (13) uses the function $f\left(x\right)=\frac{R\cdot x}{1+x\left(R-1\right)}$ to guarantee that the sensitivity never exceeds 1.0. While previous work has described the use of intermediate nodes to express the aggregate sensitivity of correlated findings (Yu et al., 1988; Nikovski, 2000; Velikova et al., 2014), we are unaware of other successful attempts to express the diagnostic power and sensitivity of the intermediate node as independent finding sensitivities for the disorder per Equation (9). This process is critical to avoid semantic disjunction in MDM computations. To see why this is the case, consider dependent disorders *D*_1_ and *D*_2_. Suppose findings *F*_1_ and *F*_2_ are relevant to both disorders, but are only conditionally *dependent* with respect to *D*_1_. If we were to replace *F*_1_ and *F*_2_ by an xopathy node *Xop*(*F*_1_, *F*_2_) as a finding of *D*_1_, then the disorder cluster {*D*_1_, *D*_2_} would have 3 findings instead of 2, thus creating semantic disjunction and rendering the equivalent sensitivities incorrect.

### Finding Contingency Hierarchies

The finding contingency hierarchy represents a formalization of the “drill-down” conventions of the medical interview. The top finding (e.g., “chest pain”) is usually followed by more specific findings like *quality or character* of the pain (e.g., sharp, dull, stabbing, burning, pressing), exacerbating factors (e.g., cough or exercise), relieving factors (e.g., drinking water or sitting up), etc. For many “top level” findings like chest pain or skin rash there may be tens of additional secondary or contingent findings that need to be discerned to obtain a clear picture of the disease state.

We say that finding *F*_*c*_ is *contingent* on *F*_*p*_ (and *F*_*p*_ is a *prerequisite* of *F*_*c*_) if *F*_*c*_ has no meaning unless *F*_*p*_ has been discerned. Usually, *F*_*c*_ won't have any meaning unless *F*_*c*_ takes on specific state(s). For binary findings, this condition is always that the prerequisite finding must be positive. For example, we can't ask about chest pain quality if the patient has denied chest pain. Note that a prerequisite finding may have multiple contingents, and that a contingent finding may also have multiple prerequisites. Furthermore, contingencies may be chained or nested to multiple levels.

In some cases the contingency chain must be queried in a specific order to create a coherent interview that makes sense to the patient. For example, if the patient complains of a skin lesion, we cannot ask “How deep is the ulcer?” unless we first determine that the lesion is, indeed, an ulcer. Similarly, if the patient complains of abdominal pain, there is no point asking “Is the pain relieved by antacids?” (suggests a peptic ulcer) unless we first discern that the pain is located in the upper abdomen. Similarly, we cannot ask “Which came first, the abdominal pain or the nausea & vomiting?” until we have discerned that both findings were reported.

Finding contingency chains present an interesting dilemma, namely, what probability to assign to contingent findings whose prerequisites are irrelevant to an index disorder. To illustrate this scenario, suppose the patient presents with 2 positive findings, *F*_1_ and *F*_2_ and that there are 3 contending disorders, *D*_1_, *D*_2_, and *D*_3_. Suppose *F*_1_ is relevant to all 3 disorders and *F*_2_ is relevant only to *D*_1_ and *D*_2_. For simplicity assume all disorders have the same incidence, all findings have a sensitivity of 0.3 to all relevant disorders, and that all findings have a noise sensitivity of 0.02. The relative probabilities of the disorders at this point are $P\left(D_{1}\right)/P\left(D_{2}\right)/P\left(D_{3}\right)=0.3^{2}/0.3^{2}/0.3\cdot 0.02$. The relative probability of *D*_3_ has decreased by approximately an order of magnitude. Now suppose *F*_2_ has contingent finding *F*_21_that is positive in the patient, and only relevant to *D*_1_. The updated relative probabilities are $P\left(D_{1}\right)/P\left(D_{2}\right)/P\left(D_{3}\right)=0.3^{3}/0.3^{2}\cdot 0.02/0.3\cdot 0.02^{2}$. The decrease of *P*(*D*_2_) relative to *P*(*D*_1_) seems justified, because given *F*_2_, *D*_1_ matches the finding pattern better than *D*_2_. However, *D*_3_ has essentially been punished twice for not explaining the prerequisite finding. Each time we query another finding in the *F*_2_ contingency chain the relative probability of *D*_3_ will decrease by the probability factor 0.3/0.02, and very quickly *D*_3_ will be discarded from consideration. We use the term “*don't care” finding* to mean a positive contingent finding for a prerequisite that is irrelevant to the index disorder. In our example, *F*_21_ is a “don't care” condition for *D*_3_. We further stipulate that the relative probability of a disorder should be minimally impacted by its “don't care” findings. The solution we implemented was to derive a weak positive sensitivity to “don't care” findings.

### The MidasMed Diagnostic Engine and Web App

The diagnostic engine is implemented as a web server that receives stateless diagnostic requests from a client, and returns a response consisting of a probability ranked differential diagnosis and a ranked list of the best next findings to discern. The first step is to generate a list of all valid diagnoses that explain at least one abnormal patient finding. The disorder list is used to create a dynamic sparse BN. It is sparse, because it contains only valid diagnoses for the given request. As described earlier, the conditional probabilities for each parent disorder are represented as statistical aggregates of the children. Note that there is no need to compute the entire finding conditional probability distribution, only the probability of the patient value. A recursive computation is then initialized with the ancestor disorders of each subtype family. MDM computations are applied, and the disorders are placed in a stack and ranked by descending relative probability. The Child Better than Next heuristic is then applied recursively (starting at the top of the disorder stack), by replacing the next qualified parent and all its siblings by all their children, updating the relative probabilities, and resorting the stack. Note that only the MDM cluster containing the parent(s) needs to be recomputed with each replacement. The resulting final differential diagnosis offers the user the appropriate diagnostic subtype specificity for the known findings.

**Figure 4:**
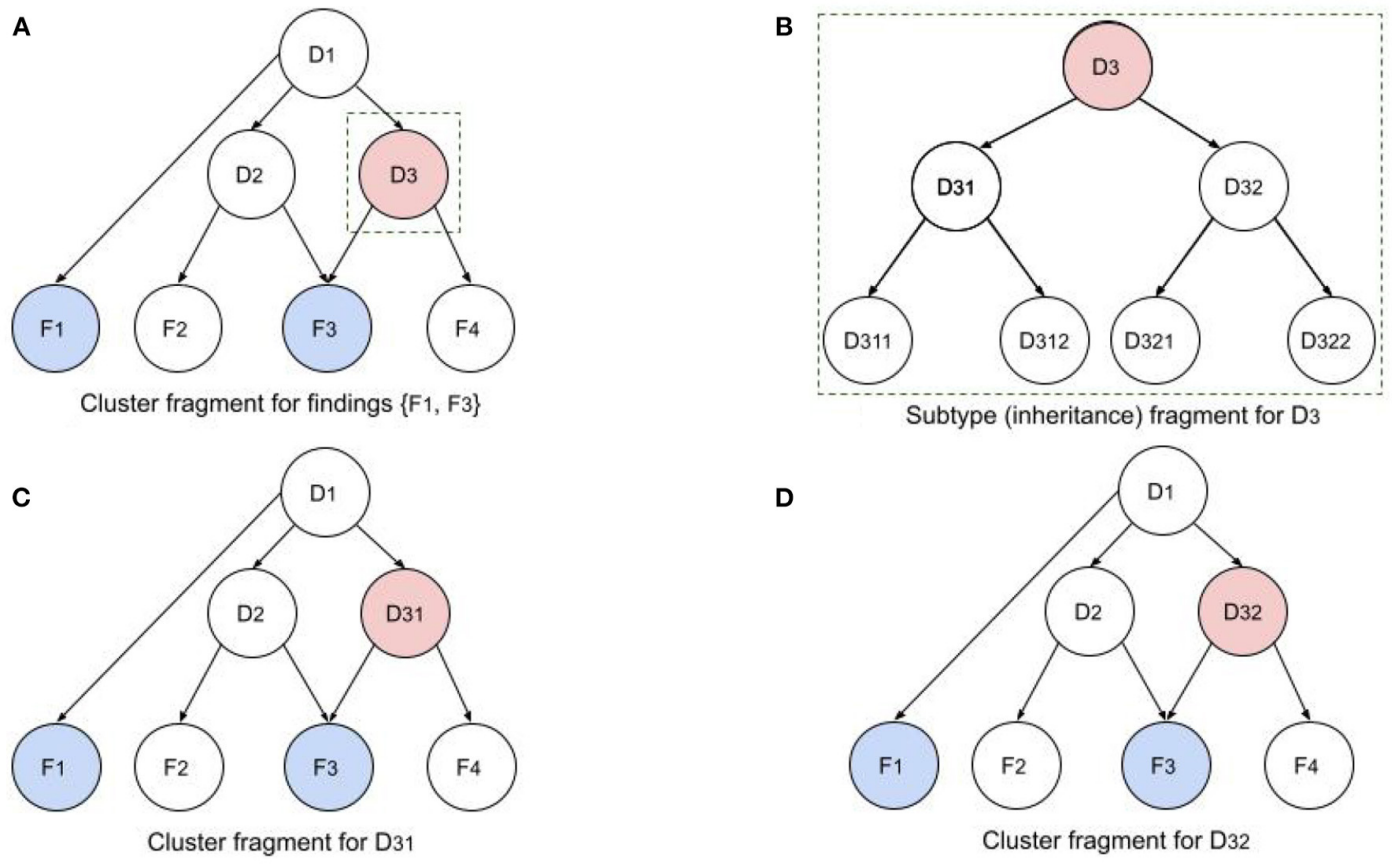
Illustration of recursive BN computations for disorder cluster and subtype fragments. **(A)** Cluster fragment for patient findings *F*_1_ and *F*_3_ and disorder subtype ancestors. **(B)** Subtypy tree for disorder *D*_3_. **(C,D)** *D*_3_ in original network has been replaced by its children *D*_31_ and *D*_32_ to compute the cluster probabilities with the two children.

Figure 4 illustrates a fragment of a single iteration in this recursive process. Figure 4A shows a cluster fragment for patient findings *F*_1_ and *F*_3_. Note that *F*_3_ is relevant to both *D*_2_ and *D*_3_, so it will require an equivalent sensitivity for configurations in which both disorders are present. In the next iteration (if the Child Better than Next criterion is satisfied) *D*_3_ will be replaced by children *D*_31_ and *D*_32_. In the following iteration *D*_31_ and *D*_32_ (siblings) will be replaced by all their children (*D*_311_, *D*_312_, *D*_321_, and *D*_322_). Note that the network in Figure 4A depicts causality (e.g., *D*_1_ causes *D*_2_ and *D*_3_), while the network in Figure 4B depicts disorder subtypes (e.g., *D*_3_ is a supertype of *D*_31_ and *D*_32_). Subtypes of a single parent (siblings) are considered mutually exclusive, so *P*(*D*_31_) is computed using the configurations of the cluster in Figure 4C. However, the probabilities of the dependent disorders (*D*_1_ and *D*_2_) are computed from the configurations of both Figures 4C,D, by summing the probabilities of all configurations in which they appear. Similarly, in the next recursion, configurations will be computed with *D*_311_, *D*_312_, *D*_321_, and *D*_322_.

The innovations described above combine to produce a nuanced approach to diagnosis that we assert results in substantially greater accuracy than existing solutions in that the differential diagnosis probabilities are more consistent with the evidence available to support them. We further assert that with diagnostic guidance based on Bayesian probabilities, heuristics, and estimated costs, the differential diagnosis converges to the correct diagnosis more efficiently, potentially translating into time and cost savings.

Our prototype system (MidasMed) currently recognizes a limited subset of 200 common adult primary care disorder subtype families (760 total diagnoses) spanning a variety of systems (respiratory, dermatology, neurology, musculoskeletal, etc.), and 4,000 findings (We estimate these encompass approximately half of the disorders a competent primary care physician should be able to recognize.). The semantic network is defined using statistical and logical analysis of epidemiological data, case series, journal articles, textbooks, and other online resources.

MidasMed includes a user-friendly web app for both patients and clinicians using dual vocabularies and default application settings for the two distinct user groups. For example, by default patients and lay caregivers are presented only with history questions in lay terminology, while professional users are asked all finding types (including exam and test results) using professional terminology. The user interface is interactive, and is designed to give the user maximum flexibility and control. Throughout the encounter, patient findings can be augmented, edited or deleted. The user can choose from 3 ways of entering new findings to refine the initial differential:

1. Search: The user selects her own findings from a global findings list.
2. Guide Me: MidasMed asks a short series of the best next questions to discern.
3. Drill Down: The user selects a disorder from the differential diagnosis to view, rank, and select undiscerned findings for that disorder. This allows the user to focus on a condition of particular concern due to urgency or severity, and answer the questions that will most efficiently rule it in or out.

### Experimental Paradigm

In this research we compare the performance of MidasMed to that of physicians and six other publicly accessible online diagnostic aids: Ada, Babylon, Buoy, Isabel, Symptomate, and WebMD. To facilitate a comparison with previous studies, we used a set of publicly available case vignettes (Semigran et al., 2015) that were tested on 23 symptom checkers in 2015, physicians (Semigran et al., 2016) and on three physicians and the Babylon DA in 2020 (Baker et al., 2020). The vignettes are available online in the format of Table 1. (See Table 5 in the Supplementary Materials for a complete list of vignettes and also a link to the vignettes file).

**Table 1:** Sample vignette.

**Diagnosis**	**Vignette**	**Simplified (*added symptoms*)**
**Requires emergent care (*****n*** **= 15)**		
Appendicitis	A 12-year-old girl presents with sudden-onset severe generalized abdominal pain associated with nausea, vomiting, and diarrhea. On exam she appears ill and has a temperature of 104°F (40°C). Her abdomen is tense with generalized abdominal pain, nausea, tenderness and guarding. No bowel sounds are present.	12 y/o f, sudden onset severe abdominal pain, nausea, vomiting, diarrhea, T = 104

As in the previous studies (Semigran et al., 2015; Baker et al., 2020), we used only the information from the “Simplified (*added symptoms*)” column of the vignette file, and excluded vignettes based on conditions on which MidasMed had not yet been educated (in conformance with the methodology of Baker et al., 2020). This resulted in a test set of 30 vignettes, the same number used in Baker et al. (2020). We note that none of the vignettes had been used in the training, education or parameterization of MidasMed.

We regarded the diagnosis presented in the “Diagnosis” column of the vignette file as the true or “target” diagnosis, except in 2 cases where no final diagnosis was provided but was clearly implied (the implied diagnosis was used), and 2 cases where multiple causally linked disorders were implied by the vignette history (either implied diagnosis was accepted). We did not find descriptions of how these problematic vignettes were treated in the previous articles. These exceptional cases are clearly identified in Table 5 in the Supplementary Materials.

In two cases the diagnosis provided for the vignette seemed inadequately substantiated by the simplified vignette history in our clinical opinion. For presumed consistency with the previously reported research, we nonetheless regarded it as the target diagnosis. These cases are also identified in the Supplementary Materials (Table 5).

MidasMed is an incomplete prototype, and therefore has not been publicized or promoted, but is publicly accessible (for a limited time) for evaluation and feedback at midasmed.com, and the vignette cases created for this article are publicly accessible *via* the application for anyone to view and experiment with (see instructions in the Supplementary Materials). At this writing, MidasMed recognizes only 200 adult disorder families. A complete list of supported diagnoses can be found in the app at midasmed.com from the Options (hamburger) menu.

For this study we used all of the adult vignette cases from the source file on which MidasMed has been educated, plus three pediatric cases for which the presentation is very similar to that in adults. Since MidasMed only accepts patient ages ≥ 18, the ages of the three pediatric patients were transposed to 18 years.

All the other DAs evaluated are publicly promoted as diagnostic aids for the general public. (One limits the age to ≥ 16, for which the age of the two younger patients was also transposed to that minimum age). Since none of the vignettes are based on rare disorders, we assumed the other DAs to be capable of recognizing all the target diagnoses.

The data for physicians and the Babylon DA were taken from Baker et al. (2020), and were not independently replicated in this study. For each of the other diagnostic assistants one of us (D. Jones, MD, board certified in emergency medicine with 25 years' primary care experience) entered only the “Simplified (*added symptoms*)” findings for each vignette into the online DAs (See the Supplementary Materials for links to all the DAs). Note that these simplified vignettes were designed to reflect only the history findings and observations that a patient could enter. For each DA we recorded (a) the fraction of cases for which the target diagnosis was #1 in the list of diagnoses provided; and (b) the fraction for which the target diagnosis was in the top 3 disorders of the list.

## Results

The results of our research are presented in Table 2.

**Table 2:** Performance comparison summary results for 7 DAs and physicians.

**Physician or DA**	**Vignettes tested**	**Target diagnosis ranked #1**			**Target diagnosis in the top 3**		
		**Fraction**	**Percent (%)**	**95% CI^e^**	**Fraction**	**Percent (%)**	**95% CI^e^**
Physicians^a^	90^b^	68/90^b^	75.3	65.4–84.0	81/90^b^	90.3	81.9–95.3
Ada	30	22/30	73.3	54.1–87.7	27/30	90.0	73.5–97.9
Babylon^a^	30	21/30	70.0	50.6–85.3	29/30	96.7	82.8–99.9
Buoy	21^c^	11/21	52.4	29.8–74.3	15/21	71.4	47.8–88.7
Isabel^d^	30	15/30	50.0	31.3–68.7	21/30	70.0	50.6−85.3
MidasMed	30	28/30	93.3	77.9–99.2	29/30	96.7	82.8–99.9
Symptomate^d^	30	21/30	70.0	50.6–85.3	26/30	86.7	69.3–96.2
WebMD^d^	30	20/30	66.7	47.2–82.7	28/30	93.3	77.9–99.2
All DAs	201	138/201	67.7	61.8–75.0	175/201	87.1	81.6–91.4
Top 3 DAs^f^	90	71/90	78.9	69.0–86.8	82/90	91.1	83.2–96.1

a *The Babylon and physician tests were not replicated in this study, but were transcribed from Baker et al. (2020), which used the same methodology*.

b *In the Babylon study three physicians were tested, but only percent data were reported; therefore 95% CI's were computed assuming a total of 90 vignettes (30 per doctor)*.

c *For 9 of the 30 disorders presented, Buoy gave no proposed diagnoses; only triage recommendations (e.g., “Contact a medical professional” or “Call 911!”)*.

d *Isabel, Symptomate, and WebMD are the only DAs tested both in the original paper (Semigran et al., 2015) and this study*.

e *CI intervals were computed using Clopper-Pearson exact method for binomial probability distributions*.

f *For a larger sample size to compare with physicians, we combined the top 3 DAs we tested (Ada, MidasMed, and Symptomate)*.

### Limitations Regarding Our Results

Although MidasMed aspires to be a complete diagnostic aid for both patients and clinicians, and therefore includes the physical examination and test findings required to definitively diagnose the disorders on which it has been educated, only history findings were entered in this study. The objective here was to quantify the ability to identify the correct diagnosis based on sparse patient histories, as are readily available directly from patients online.

With only 30 cases, the statistical reliability of the results is low, as reflected in the broad confidence intervals. The original study for which the vignettes were created (Semigran et al., 2015) included 45 vignettes, but only the 27 adult plus 3 pediatric disorders on which MidasMed has been educated were tested in this study, and only 30 in the study (Baker et al., 2020) that produced the physician and Babylon data reported here.

It is possible that as the breadth of disorders covered by MidasMed is increased, and the correct diagnosis must compete with a greater number of similar disorders, accuracy will decline. However, since (a) the disorders presently covered by MidasMed were selected because they are among the most common, and (b) the vignette diagnoses are mostly common disorders, adding the less common disorders is unlikely to hinder the recognition of the vignette disorders. Rather, it will be difficult (probably impossible) to correctly identify an uncommon disorder (e.g., bronchiectasis or idiopathic pulmonary fibrosis) as the most likely diagnosis based on only sparse vignette histories such as were used here, some of which contain only 3 or 4 common findings.

It was difficult to make perfectly fair comparisons of the different DAs due to differences in their user interface (UI) approaches. For example, some apps (e.g., MidasMed, Ada) offer an “unknown” option for (virtually) every follow-up question queried, making it easy to limit the information entered strictly to the items provided in the simplified vignettes. However, other DAs (e.g., Buoy, Symptomate), presented follow-up questions that required an affirmative or negative answer to proceed. In those cases (i.e., when forced to provide information not in the vignette), we attempted to err in the direction of aiding the DA under test, by answering as a typical patient with the target disorder would most likely answer. In a few cases, it was not possible to enter all history items for a specific vignette because an item was both (a) not accessible in the DAs search facility (despite trying multiple synonyms), and (b) not queried *via* follow-up questions presented by the DA.

## Discussion

Canadian physician Sir William Osler (1849–1919), “the father of modern medicine,” is known for saying, “Listen to your patient, he is telling you the diagnosis.” This message repeats in the medical school maxim, “90% of the diagnosis comes from the history, 9% from your examination, and 1% from tests” (Gruppen et al., 1988; Peterson et al., 1992). This maxim has been forgotten in today's over-stressed healthcare system. Too rushed to take a comprehensive history, doctors often compensate by ordering test panels, referring to specialists, and scheduling more follow-up visits; “Next patient, please.” Patients on the receiving end are justifiably frustrated and open to alternatives. But with the growing role of telehealth, where the ability to perform exams or order stat tests is limited, patient history should regain its role as the primary factor in the diagnostic equation. There is also a broader trend toward democratizing access to medical information, or “eHealth” *via* phone apps, wearables, and inexpensive measurement devices, giving patients more control over care options.

In this study we performed a prospective validation of a novel Bayesian diagnostic assistant (MidasMed), and compared it to five online DAs (Ada, Buoy, Isabel, Symptomate, and WebMD) and to the accuracy previously reported for the Babylon DA and physicians. MidasMed was able to identify the correct diagnosis as most likely with 93% accuracy, significantly outperforming physicians (75%) on the same vignettes (Baker et al., 2020).

We attribute the superior performance of MidasMed to a diagnostic model that moves beyond the “leaky noisy OR gate” assumption of conditional independence among the BN nodes (Henrion, 1987), and to reducing semantic overlap and disjunction that are common in the medical literature and can lead to significant distortion in estimated probabilities of the outcomes. These simple vignettes and our scoring technique did not give MidasMed credit for diagnosing co-present causally related disorders. In particular, it is noteworthy that for the two vignettes that imply the causal co-occurrence of multiple disorders, MidasMed produced estimated relative probabilities for these disorders whose sum approaches 200%, implying a high likelihood of co-occurrence (See the Supplementary Materials, for instructions to access the cases online).

It appears from our results that the accuracy of online DAs has improved significantly in the 6-year interim since the original paper (Semigran et al., 2015) evaluated the study vignettes. In that paper, the best-performing symptom checker listed the target diagnosis first only 50% of the time, and in the top three only 67% of the time; and the average performance of 19 symptom checkers in that study for the top 1 and top 3 was only 34 and 51%, respectively. Whereas in this study, the best performance was 93% (top 1) and 97% (top 3); and the average DA performance was 68 and 86%, respectively, showing significant improvement. Furthermore, in this study the performance of the top three DAs combined was 78.9% (top 1) and 91.1% (top 3), comparing very favorably with physicians (75.3 and 90.3%, respectively). Note that in the later comparison we use the 90 vignette aggregates, with similar narrower confidence intervals.

We note several differences in test methodology that may have contributed to the *apparent* accuracy improvements relative to Semigran et al. (2015) for previously tested DAs. First, in Semigran et al. (2015), all data was entered by non-clinicians, who may not have been as facile at matching symptoms to their various DA synonyms as the physician-testers in this study and in Baker et al. (2020). However, that method may give a better estimate of “read world” performance with real patients seeking diagnosis. Second, responses to “mandatory” questions (without which the interview does not proceed, but are not answered by the vignette) may have been entered inadvertently in a way that “punished” the target diagnosis, whereas in this study we explicitly answered such questions to favor the target diagnosis. Third, in Semigran et al. (2015) all 45 vignettes in the source file were used to test all DAs without verifying support for the target diagnosis. These factors may have contributed to the lower scores in the earlier study.

### Future Work

At this time MidasMed recognizes a limited set of disorders spanning all organ systems, but lacks comprehensive coverage for any specific system. To complete our technology validation, we plan next to expand its education to *in-depth coverage* of a major organ system (e.g., gastrointestinal and hepatobiliary disorders), and verify that (a) it continues to recognize *most* disorders as the likely diagnosis based on history alone, (b) it recognizes *all* disorders with high accuracy when exam findings and tests are included, and (c) it guides the user efficiently from the initial differential to the definitive diagnosis by optimizing a preset criterion (e.g., diagnostic utility-to-cost ratio). When sufficient data has been acquired, we will apply statistical reliability measures (e.g., Hilden et al., 1978) to assess the confidence and diffidence of the DA's probability estimates.

Although the goal of this paper was limited to the comparison of the diagnostic accuracy of currently available online diagnostic assistants using standardized vignettes, we hope in future work to present our diagnostic innovations in greater detail, and to explicitly measure and compare the accuracy contribution of individual algorithmic innovations (e.g., our modeling of dependencies among findings, modeling of subtypy relationships among disorders, use of continuous probability distributions, etc.).

In this work, to facilitate an apples-to-apples comparison with prior results, we tested on a small set of case vignettes previously tested in Semigran et al. (2015, 2016); Baker et al. (2020). We hope in future work to test across multiple DAs using larger sets of test cases.

## Data Availability Statement

The original contributions presented in the study are included in the article/Supplementary Material. Further inquiries can be directed to the corresponding author/s.

## Author Contributions

DJ contributed to the study design and data analysis, entered the symptoms into the diagnostic assistants, and contributed to the writing of the paper. AJ designed the diagnostic software involved, participated in the study design and data analysis, and contributed to the writing of the paper. Both authors contributed to the article and approved the submitted version.

## Conflict of Interest

AJ and DJ are with Eureka Clinical Computing, the creator of the MidasMed DA.

## Publisher's Note

All claims expressed in this article are solely those of the authors and do not necessarily represent those of their affiliated organizations, or those of the publisher, the editors and the reviewers. Any product that may be evaluated in this article, or claim that may be made by its manufacturer, is not guaranteed or endorsed by the publisher.
