# Supplementary material for: A Novel Bayesian General Medical Diagnostic Assistant Achieves Superior Accuracy With Sparse History: A Performance Comparison of 7 Online Diagnostic Aids and Physicians
Source: Front Artif Intell. 2022 Jul 22;5:727486. doi: 10.3389/frai.2022.727486 (PMC9355422; doi:10.3389/frai.2022.727486)
Supplement: Supplementary file 1 [file Table_1.pdf]

SUPPLEMENTARY MATERIALS

Diagnostic power measures

	Disease (D)		
**Finding (F)**	**D+**	**D-**	**total**
**F+**	A	B	A+B
**F-**	C	D	C+D
**total**	A+C	B+D	

Table 3: Number of patients with and without a disease and with or without a finding. E.g., *A* is the number of patients with the disease in whom the finding is present, and *B* is the number of patients without the disease in whom the finding is present.

CAVEAT: In using these disease and non-disease statistics and the formulas derived from them in Table 4 below, it's critical to know the source of *D-*, the population without the disease. It could range from the general population without the disease to a narrowly defined set of ill patients with specific symptoms, as is typical for many studies reporting test statistics.

Test	Equation per Table 3 (above)
sensitivity (S)	*A/(A+C)*
specificity (S_p)	*D/(B+D)*
positive predictive value (PPV)	*A/(A+B)*
negative predictive value (NPV)	*D/(C+D)*
positive likelihood ratio (LR^+)	$\frac{A}{B} \cdot \left(\frac{B+D}{A+C}\right) = \frac{S}{1-S_p}$
negative likelihood ratio (LR^-)	$\frac{C}{D} \cdot \left(\frac{B+D}{A+C}\right) = \frac{1-S}{S_p}$
diagnostic odds ratio (DOR)	$\frac{A \cdot D}{B \cdot C} = \frac{LR^+}{LR^-}$
Probability factor	$\frac{A}{(A+C)} \div \frac{(A+B)}{(A+B+C+D)}$

Table 4: Measures to quantify the discriminating power of a finding.

The Diagnostic Assistant web links:

The online diagnostic assistants or symptom checkers reviewed in this article can be accessed at the following web addresses. (These are liable to change over time.)

Ada: www.sutterhealth.org/health/symptom-checker

Babylon: www.babylonhealth.com/us/what-we-offer/chatbot (Requires iPhone or Android app)

Buoy Health: www.buoyhealth.com/symptom-checker/

Isabel: symptomchecker.isabelhealthcare.com/suggest_diagnoses_advanced/landing_page

MidasMed: midasmed.com (See instructions below to view case vignettes in MidasMed.)

Symptomate: https://symptomate.com/

WebMD: https://symptoms.webmd.com/

To view the vignette cases with their differential diagnosis lists as analyzed by MidasMed:

(For any problems or issues with the online demo, please contact the corresponding author. The public midasmed.com prototype will be maintained for a limited time, after which a private demo may be available by contacting the corresponding author.)

1) Go to https://midasmed.com

2) Select "I'm a healthcare professional" on the Welcome dialog.

3) Click "Sign In" on the titlebar and log in with the following credentials:

Email: demo21@mail.com

Password: demo21

You will then be presented with the Patient list showing the (fictitious) names of the vignette patients. (See the full list of vignette cases and names in Table 5, below.)

4) Select any patient whose case you'd like to see, and click Next.

5) Select the single encounter for this patient and then click Next.

On a large screen device you'll see the patient findings and the MidasMed-generated differential diagnosis side-by-side, while on a mobile device you'll see one list at a time.

You may experiment with the cases (add, edit, or delete findings) ad lib, but for this demo any changes cannot be saved. You can always create your own account and save any encounters you create.

To provide specific feedback on a case, click "Contact us" at the bottom of the application window, select the appropriate category, enter your feedback, and select Send.

NOTE 1: Since this research was performed, COVID-19 has become highly prevalent, and may contend strongly (and appropriately we believe) with the original diagnosis for some of the respiratory disorder vignettes.

NOTE 2: If diagnosing new cases de novo, bear in mind that MidasMed has been educated on only about half of the disorders a competent primary care physician should recognize. To view the covered diagnoses, select "supported diagnoses" from the app's hamburger menu.

List of case vignettes (with age, gender, fictitious names used by MidasMed, and notes):

The complete list of 45 vignettes was provided in an appendix to (Semigran, 2015), and is available at:
bmj.com/content/bmj/suppl/2015/07/07/bmj.h3480.DC1/semh025489.ww1.pdf

Of the 45 vignettes, we excluded 10 pediatric cases, and 5 cases corresponding to diagnoses MidasMed does not currently recognize. The 30 vignettes used in this study are listed in Table 5, below. Only the sparse history findings in the "Simplified (*added symptoms)*" column of the source file were used (those findings a typical patient could be expected to enter).

In our scoring, we accepted all standard synonyms (e.g., "glandular fever" for "mononucleosis") and subtypes (e.g., "viral meningitis" for "meningitis") of the file diagnosis as correct answers. In two cases no diagnosis was provided, only the symptom "back pain," but for each of those cases a specific diagnosis was clearly clinically implied by the history, and that was used as the target diagnosis. Although only a single diagnosis was given for each vignette, two were clear-cut cases of causal disorder combinations (one case of deep venous thrombosis *plus* pulmonary embolism, and the second a case of allergic rhinitis *plus* allergic conjunctivitis). In these cases, either implied diagnosis was accepted as correct. The three cases with age < 18 in the vignette file were entered into MidasMed with age 18, the minimum age required to use the app. These exceptions and anomalies are noted for the specific cases below. (How these anomalies were handled in (Semigran, 2015) and (Baker, 2020) was not explained in those papers.)

Table 5: List of case vignettes.

Vignette diagnosis, age and gender	MidasMed patient name	Notes
asthma<br>27 year-old female	Carolyn Asthma	
COPD flare<br>67 year-old female	Cathy Cope	
deep vein thrombosis<br>65 year-old female	Trina Venus	
heart attack<br>64 year-old Chinese male	Albert Chen	
kidney stones<br>45 year-old white male	Kyle Kidney	
meningitis<br>18 year-old male student	Manjeet Patel	
pneumonia<br>65 year-old male	Neal Newman	
pulmonary embolism<br>65 year-old male	Elmer Embolus	The file diagnosis is PE, but this is clinically an obvious case of *both* deep venous thrombosis (causal disorder) *and* pulmonary embolism (complication), so we gave credit for either diagnosis as the target diagnosis.
stroke<br>70 year-old male	Sylvester Strunk	
acute pharyngitis<br>24 year-old male	Daniel Shore	
acute sinusitis<br>35 year-old female	Suzy Sinus	
Back pain<br>35 year-old male	Bret Beckman	No final diagnosis was provided for this case, only the symptom "back pain," but lumbar disk herniation is clinically implied by the vignette, so was regarded as the target diagnosis.
cellulitis<br>45 year-old male	Bob Johnson	
COPD flare (milder)<br>56 year-old female	Sandra Smoker	
influenza<br>30 year-old female	Francis Warner	
mononucleosis<br>16 year-old female high school student	Molly Mono	Entered as an 18 year-old. (Symptoms are similar for teenagers and young adults.)
peptic ulcer disease<br>40 year-old male	Bjorn Peptix	
shingles<br>77 year-old male	Sam Shingler	
urinary tract infection<br>26 year-old female	Ursula Underhill	
acute bronchitis	Brenda	

34 year-old female	Ballinger	
acute bronchitis<br>61 year-old female	Lisa Lawrence	
acute conjunctivitis<br>14 year-old boy	Bobby Bradford	Entered as 18 year-old. (Symptoms are similar for teenagers and young adults.)
acute pharyngitis<br>26 year-old male	Elvis Loughlin	The history includes only 2 days of sore throat, headache, cough, no fever. For the sparse symptoms of this vignette, we don't think a definitive diagnosis of "acute pharyngitis" is warranted, since pharyngitis, per se, doesn't usually cause headache and cough. Other common diagnoses (e.g., common cold, acute bronchitis) seem at least as likely. Nonetheless, for consistency with previous studies "acute pharyngitis" was regarded as the target diagnosis.
allergic rhinitis<br>22 year-old male student	Ted Titchner	Although "allergic rhinitis" is given as the file diagnosis, this is a clear-cut case of simultaneous "allergic rhinitis" *plus* "allergic conjunctivitis," so either was accepted as the target diagnosis.
back pain<br>38 year-old male	Brad Baker	No diagnosis was provided for this vignette except the symptom "back pain," but this was in the "self-care appropriate" section of the file, in the context of lumbar pain following heavy lifting, and with neurological symptoms denied, so we assumed the intended diagnosis to be "nonspecific low back pain," "mechanical low back pain" or "lumbar strain," and all synonyms thereof were accepted as correct.
Candidal yeast infection<br>40 year-old monogamous, married female	Mona Monilia	
eczema<br>12 year-old female	Zema Peterson	Entered as 18 year-old. (Symptoms are similar for adolescents and young adults.)
stye<br>30 year-old male	Pete Style	
viral upper respiratory infection<br>56 year-old male	Yuri Nogavitch	With 6 days of non-productive cough, nasal congestion with green discharge, and fever of 100.8 F, we regard "bacterial sinusitis" as being at least as likely as "viral upper respiratory infection" because significant fever six days into an uncomplicated viral URI in an adult is unusual. Nonetheless, for consistency with prior studies we regarded "viral upper respiratory infection" as the target diagnosis.
viral upper respiratory infection<br>30 year-old male	Vern Resper	

Table 5: List of case vignettes.
